# Right Cervical Aortic Arch and Aberrant Subclavian Artery Causing Vascular Ring Formation

**DOI:** 10.7759/cureus.91802

**Published:** 2025-09-07

**Authors:** Keshav Nandakumar, Matthew L Hovancsek, Randy Richardson

**Affiliations:** 1 School of Medicine, Creighton University School of Medicine, Phoenix, USA; 2 Department of Radiology, Creighton University School of Medicine, Phoenix, USA

**Keywords:** aberrant right subclavian artery (arsa), cervical aortic arch, congenital vascular abnormality, severe tracheal compression, vascular ring

## Abstract

This case report describes a congenital anomaly involving a right cervical aortic arch and an aberrant subclavian artery (ASA) leading to vascular ring (VR) formation. This anomaly is characterized by the presence of the aortic arch in the cervical region rather than the thoracic cavity. The ASA can contribute to the formation of a VR encircling the trachea and esophagus, potentially causing severe respiratory complications. Early diagnosis and effective management in pediatric patients are crucial, given the life-threatening risk of respiratory compromise. In this case, the patient was diagnosed prenatally and initially exhibited only mild respiratory symptoms. Over time, however, the patient developed breathing difficulties and recurrent infections secondary to the VR, which prompted bronchoscopy, microlaryngoscopy, and subsequent surgical intervention. The VR was surgically corrected through aortic uncrossing and reimplantation of the right subclavian artery. Fewer than five prior reports have documented a similar case, underscoring the importance of promptly recognizing the symptoms of a potential vascular anomaly to preserve airway integrity. This report contributes to establishing surgical criteria and intervention strategies, thereby enhancing preparedness for managing similar congenital cardiac abnormalities in the future.

## Introduction

The presence of a cervical aortic arch (CAA) is a rare congenital vascular abnormality characterized by the aortic arch arising in the cervical region rather than in the thoracic cavity. When a CAA is accompanied by an aberrant right subclavian artery, it can result in the formation of a vascular ring (VR). A VR is an anatomical encirclement, often involving vessels that partially or completely surround the trachea and esophagus, which can lead to life-threatening airway compression [1]. VRs most commonly form at the level of the thoracic inlet, where the vessels in this region compress the trachea and esophagus. In addition, an aberrant subclavian artery (ASA) can be associated with the development of a subclavian aneurysm due to dilation of the diverticulum of Kommerell, an embryonic remnant of the fourth dorsal aortic arch, at its branching site from the aortic arch.

In normal fetal development, the aortic arch arises from the left ventricle, arches over the left main bronchus, and then descends into the thoracic region. The aortic arch typically gives rise to three branches: the brachiocephalic trunk, the left common carotid artery, and the left subclavian artery. The brachiocephalic trunk then gives rise to the right subclavian artery and the right common carotid artery. Under normal anatomy, none of these vessels pass posterior to the esophagus, so there is no risk of VR formation or subsequent compression, and the aortic arch remains confined to the thoracic level. In the present case, however, the embryological abnormality occurred because the proximal and middle segments of the right subclavian artery regressed, while the distal portion connected distally to the left subclavian artery. This anomaly caused the right subclavian artery to course posterior to and compress the esophagus [2].

This report describes a patient with a CAA and an aberrant right subclavian artery that completely encircled the trachea and esophagus, resulting in severe compression. This congenital abnormality is extremely rare, with fewer than five reported cases identified in a PubMed literature search. Vascular abnormalities such as this are important to document and study, as their rarity can lead to misdiagnosis and delayed treatment, with the potential for severe complications and even death.

## Case presentation

The patient received a prenatal echocardiogram that showed a ventricular septal defect, an aortic arch anomaly, and a VR. The patient was born at 37 weeks of gestation. A postnatal echocardiogram performed on the day of birth revealed a CAA, and a subsequent dedicated CT angiography (CTA) scan confirmed VR formation without airway compression. At birth and during the neonatal period, there was no respiratory distress and no indication for surgical intervention.

At two weeks of age, the patient’s echocardiogram showed a patent foramen ovale with left-to-right shunting, a small membranous or high muscular ventricular septal defect with left-to-right shunting, and mildly elevated right ventricular pressure. A follow-up echocardiogram at seven months showed consistent findings, with an intact atrial septum.

At four years of age, the patient presented with breathing issues and was diagnosed with reactive airway disease, in addition to experiencing episodes of croup and persistent cough following frequent respiratory infections. At that time, the patient also had a developmental delay, was in speech therapy, and was undergoing evaluation for autism spectrum disorder. Family history was notable for hypertension (HTN), hyperlipidemia, and asthma, but no congenital heart disease. The patient’s maternal grandmother had died of aortic dissection. An electrocardiogram during this visit showed a normal sinus rhythm. The echocardiogram findings were consistent with prior studies, with a peak velocity in the transverse aortic arch of 1.9 m/s and a peak gradient of 15 mmHg. The gradient did not indicate left ventricular hypertrophy or HTN. The aberrant origin of the subclavian artery formed a VR. Concerns were raised about abnormal vessel positioning leading to possible esophageal compression, despite the fact that most patients are asymptomatic or only experience mild symptoms.

Three months later, the patient underwent CTA due to worsening symptoms, which showed moderate compression of the trachea at the level of the crossing left-sided brachiocephalic artery. The left main bronchus was compressed between the main pulmonary artery and the descending aorta.

At five years of age, the patient underwent bronchoscopy and microlaryngoscopy following prior diagnoses of dysphagia, tracheomalacia, and laryngomalacia, which confirmed the need for urgent surgical intervention. The right aortic arch and aberrant right subclavian artery were surgically corrected through reimplantation of the right subclavian artery and aortic uncrossing [3]. The procedure involved establishing cardiopulmonary bypass, reimplanting the right subclavian artery to the right common carotid artery, and moving the descending aorta anterior to the trachea and esophagus to relieve the VR. At discharge, the patient’s medications included furosemide, albuterol, and acetaminophen.

The postoperative course was uncomplicated, and the patient was discharged. One week after surgery, the patient was afebrile, showed improved eating, and continued to be followed by the surgical team, who were pleased with the outcome.

**Figure 1:**
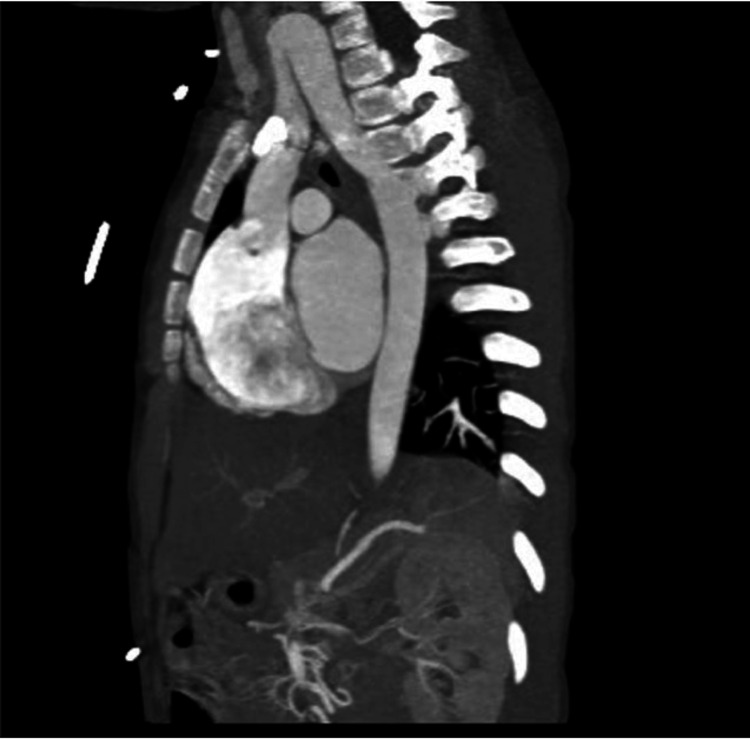
Sagittal view from chest CTA CTA, CT angiography

**Figure 2:**
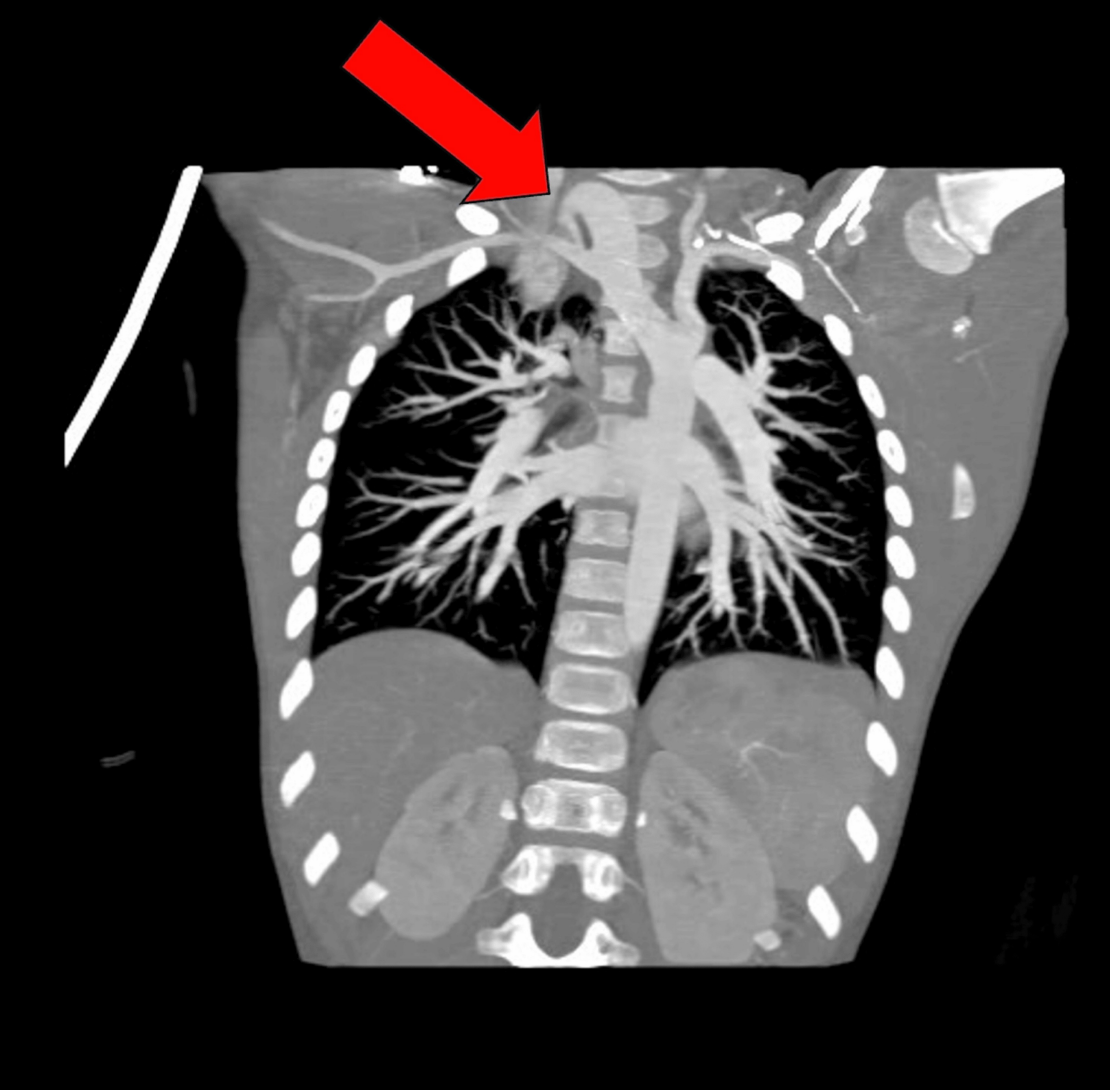
Coronal view from chest CTA The red arrow indicates the CAA on the right side of the body, contributing to VR formation. CAA, cervical aortic arch; CTA, CT angiography; VR, vascular ring

**Figure 3:**
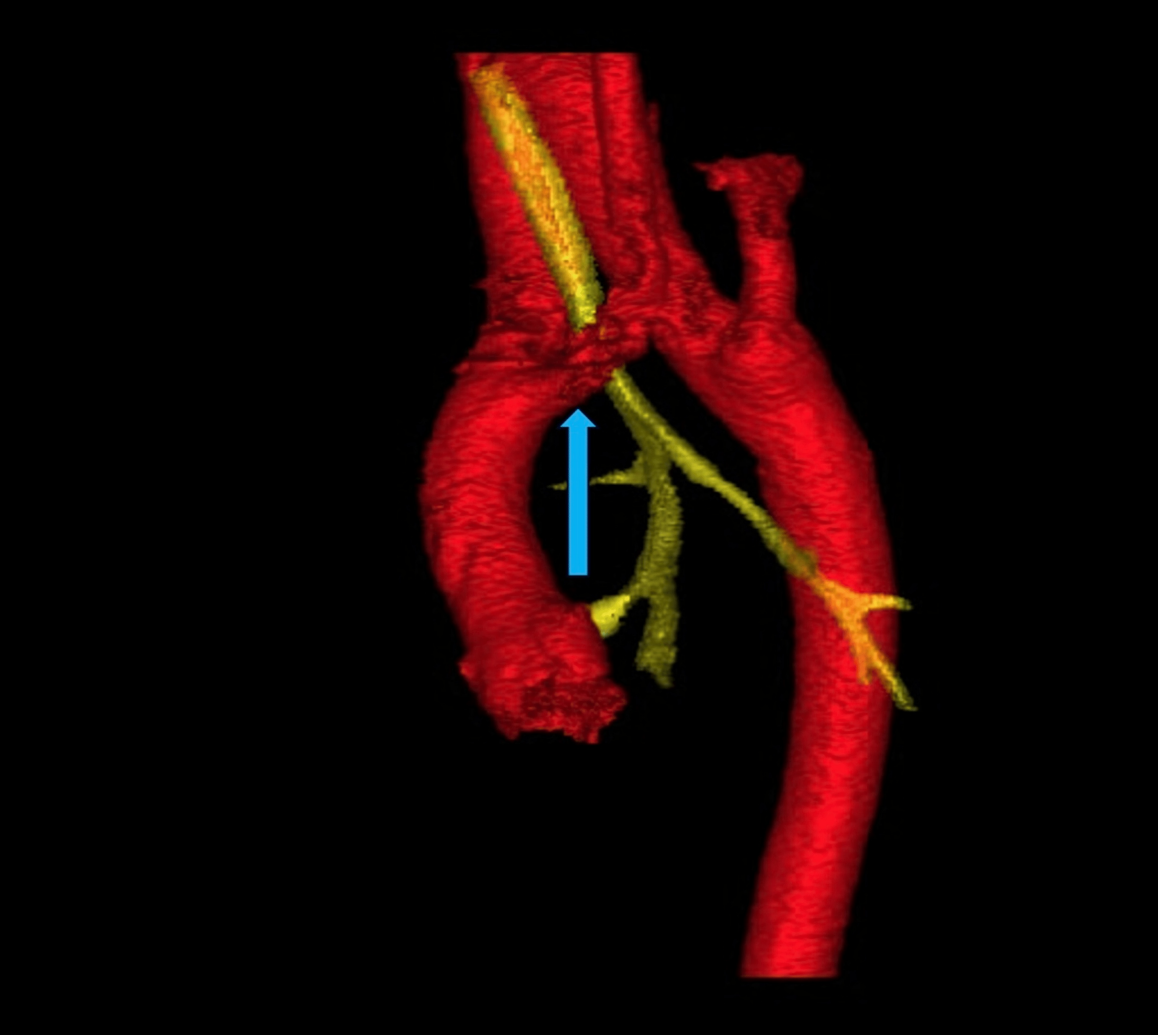
Volume-rendered 3D model from chest CTA demonstrating the VR (red) compressing the trachea (yellow) in the posterior view The blue arrow highlights the ASA forming the VR. ASA, aberrant subclavian artery; CTA, CT angiography; VR, vascular ring

**Figure 4:**
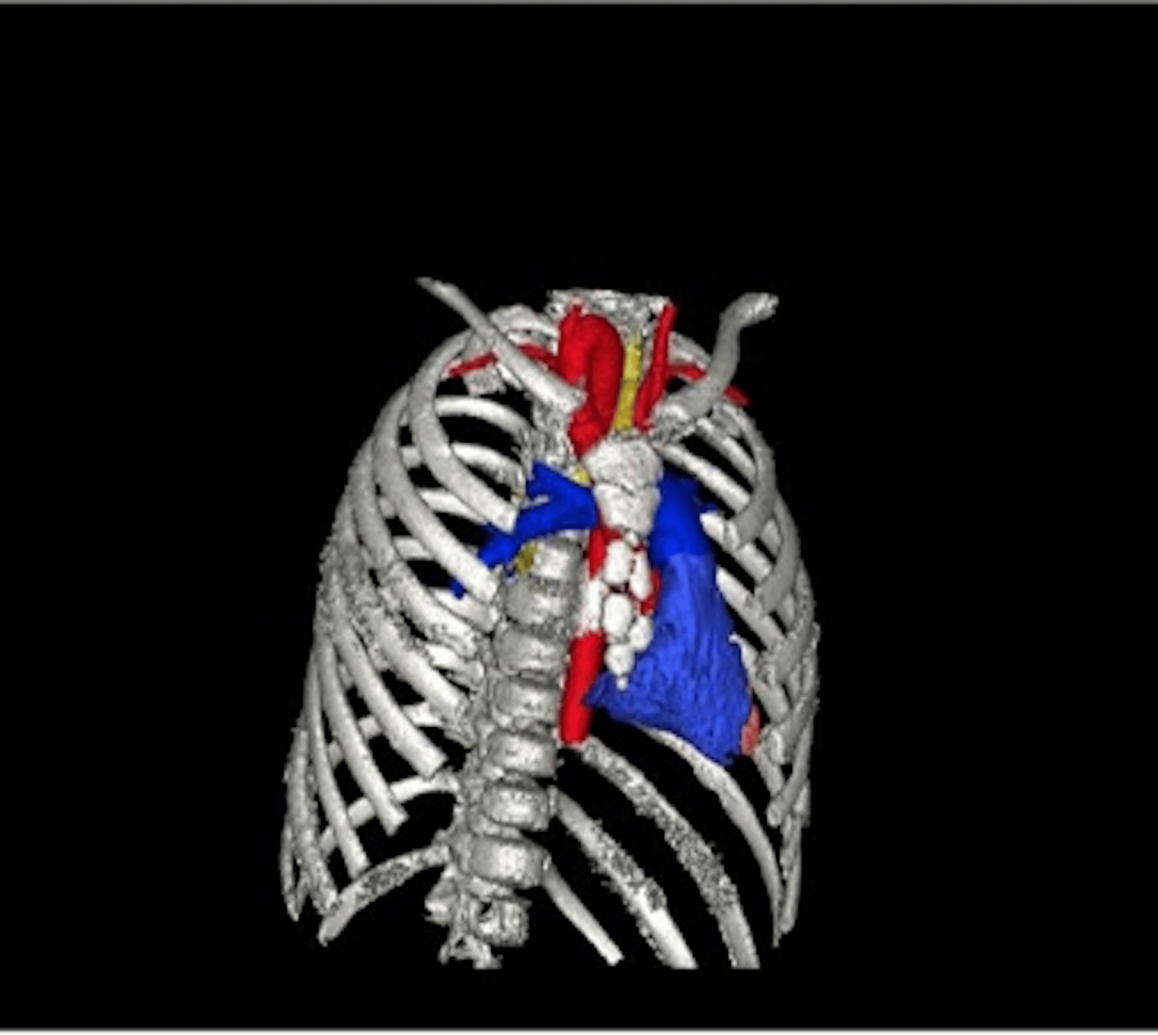
Volume-rendered 3D model from chest CTA showing the spatial orientation of the abnormalities within the rib cage in the anterior view CTA, CT angiography

The anatomical abnormalities were visualized in the patient’s scans and volume-rendered 3D models derived from imaging. The right aortic arch in this patient arose in the cervical region, rather than in the thoracic region, as in typical anatomy. The aortic arch can be seen in Figure 1 and is indicated by the red arrow in Figure 2, where it extends out of the thoracic inlet and into the neck. An associated physical examination finding was a palpable aortic arch beat above the right clavicle. The ASA in this patient arose from the descending aorta rather than the brachiocephalic trunk, as is typical. The ASA passed posterior to the esophagus and contributed to the development of a VR (Figure 3). The VR encircled both the trachea and esophagus (Figure 3 shows only the trachea for clarity), causing compression and potentially fatal respiratory complications. Symptoms of esophageal compression due to the anomalous vessel were more common than symptoms of tracheal compression [4]. Figure 4 shows the full set of anatomical abnormalities and their spatial orientation within the rib cage, illustrating the unique anatomy of this patient.

## Discussion

The case presented above highlighted a rare congenital vascular anomaly, necessitating further documentation to inform treatment options. A CAA is a rare congenital defect with an incidence of fewer than 1 in 10,000 live births [5]. The greatest concern in this case was the patient’s rapidly worsening breathing caused by the VR, which led to tracheoesophageal compression, along with an inferred atypical cardiac murmur due to mild blood flow acceleration.

Common symptoms of a VR include stridor, recurrent respiratory infections, and dysphagia. Diagnostic investigations can include a chest X-ray to demonstrate tracheal compression, echocardiography to identify the CAA, and CTA to localize the ASA. Prenatal diagnosis can be achieved with fetal echocardiography; however, most cases are diagnosed postnatally, either after symptom onset or incidentally on imaging. Differential diagnoses include asthma, bronchiolitis, tumors compressing the esophagus, and esophageal stricture, all of which can present with similar clinical features. In this patient’s case, treatment decisions were guided by the presence of symptoms.

The surgical intervention involved correcting the right aortic arch and aberrant right subclavian artery through reimplantation of the right subclavian artery and aortic uncrossing. Surgical correction of an ASA has been associated with low morbidity and mortality, excellent primary patency, and significant symptom relief [6]. This approach aligned with the established treatment for patients with similar vascular anomalies, aiming to alleviate respiratory symptoms and reduce further complications. ASA anatomy can also be associated with a subclavian artery aneurysm, for which hybrid surgical repair (a combination of open and endovascular techniques) has been reported as a less-invasive alternative for proximal subclavian artery aneurysm [7].

In adults, two common procedures are performed to repair aberrant subclavian arteries. The first is carotid-subclavian transposition, which involves redirecting the subclavian artery by anastomosing it to the carotid artery [8]. This bypasses the aberrant segment and can relieve tracheal compression. The second approach is Kommerell diverticulum resection, in which the dilated portion of the aorta at the origin of the subclavian artery is removed to prevent aneurysm rupture [8].

The prognosis of a VR is generally favorable after successful relief of compression. Early intervention is vital to prevent irreversible complications such as tracheomalacia, bronchiectasis, and chronic feeding difficulties, while also alleviating respiratory symptoms. Surgical intervention during early childhood may also promote normal tracheal growth and reduce the risk of long-term complications, although rare cases of persistent respiratory problems have been reported [9,10]. In this case, intervention occurred shortly after symptom onset, and postoperative scans confirmed the absence of airway compression and successful repair of the ASA.

An interdisciplinary approach was critical for early diagnosis, management, and intervention, ensuring high-quality care for this complex anatomical abnormality. The patient’s care team included obstetricians, radiologists, cardiologists, and cardiothoracic surgeons. Advanced imaging techniques facilitated early diagnosis and supported surgical planning and preparation. The management of this patient reflected the gold standard of care for rare congenital anomalies.

Although 3D modeling was not used preoperatively, this case highlighted its potential benefits. The software used for 3D modeling was GE Advantage Workstation version 4.6. The creation of 3D models allows visualization of spatial relationships within a patient’s unique anatomy and vascular abnormalities, thereby improving surgical planning and execution. This is particularly valuable in complex cases where dissection and resection planes must be clearly understood to ensure safety [11]. Material choice depends on the intended use: resins can provide detailed replicas for visualization, whereas pliable polymers such as silicone can be used for hands-on surgical practice, such as reimplantation techniques, which were performed in this case. Despite its advantages, 3D modeling has limitations, including cost, accessibility, and time requirements, which may restrict its use. Continued development and wider implementation of 3D modeling are expected to further enhance surgical planning and improve patient outcomes.

Giordano et al. reported a similar case involving an infant with a left CAA and an aberrant right subclavian artery causing a VR [12]. A key difference in that case was the presence of pulmonary artery stenosis, which necessitated urgent surgical intervention at 30 days of life. As in our case, reimplantation of the right subclavian artery was performed; however, the surgical team opted not to perform aortic uncrossing, instead carrying out an aortopexy and tracheopexy.

This case adds to the limited body of knowledge on the management and outcomes of rare congenital heart defects. Further research is needed to improve the generalizability of treatment strategies for these anomalies. Standardization of care is challenged by the rarity and heterogeneity of such cases, emphasizing the importance of documenting and publishing detailed reports. Future research could focus on building larger cohorts of similar cases to guide standardized management and evaluate the long-term impacts of surgical intervention, including quality of life outcomes.

## Conclusions

This case report highlights the importance of early diagnosis and imaging in providing the patient with the best possible care. In this case, management began prenatally due to the rapid identification and intervention of the anatomical abnormalities, allowing the teams time to prepare before severe airway compromise could occur. Multidisciplinary coordination across all specialties and members of the healthcare team was also essential to surgically correct the abnormalities before tracheal and esophageal compression could threaten the patient’s well-being or long-term quality of life.

This report further emphasizes the need for an individualized approach to congenital vascular abnormalities, as slight anatomical variations may necessitate different treatment plans, even among patients with similar vascular anomalies. The use of advanced imaging, 3D modeling, and 3D printing in this case demonstrates the potential of these techniques to significantly support the surgical correction process. Additional documentation of case reports and multicenter collaboration remains necessary to standardize management and establish protocol guidelines for CAA variations and VRs. Future projects that track long-term surgical outcomes and quality-of-life measures will be valuable for further refining pediatric protocols and guidelines for managing similar patients.
